# The Bacteroidales produce an *N*-acylated derivative of glycine with both cholesterol-solubilising and hemolytic activity

**DOI:** 10.1038/s41598-017-13774-6

**Published:** 2017-10-16

**Authors:** Alli Lynch, Elaine Crowley, Eoghan Casey, Rafael Cano, Rachel Shanahan, Ger McGlacken, Julian R. Marchesi, David J. Clarke

**Affiliations:** 1 School of Microbiology, University College Cork, Cork, Ireland; 2 APC Microbiome Institute, University College Cork, Cork, Ireland; 3 Department of Chemistry, University College Cork, Cork, Ireland; 4 School of Biosciences, Cardiff University, Museum Avenue, Cardiff CF10 3AX, UK and Centre for Digestive and Gut Health, Imperial College London, London, W2 1NY UK

## Abstract

The contribution of the gut microbiota to the metabolism of cholesterol is not well understood. In this study, we identify 21 fosmid clones from a human gut microbiome metagenomic library that, when expressed in *Escherichia coli*, produce halos on LB agar supplemented with 0.01% (w/v) cholesterol (LBC agar). Analysis of 14 of these clones revealed that they all share a fragment of DNA with homology to the genome of *Bacteroides vulgatus*. The gene responsible for halo production on LBC agar, named *choA*, was identified as an *N*-acyltransferase known to produce an acylated glycine molecule called commendamide. In this study we show that commendamide is capable of producing a halo on LBC agar suggesting that this molecule is solubilizing the cholesterol micelles in LBC agar. We also show that commendamide is responsible for the previously described hemolytic activity associated with the *choA* orthologue in *Bacteroides fragilis*. A functional analysis of ChoA identified 2 amino acids that are important for commendamide biosynthesis and we present phylogenetic and functional data showing that orthologues of *choA* are found only in the order Bacteroidales. Therefore, the production of commendamide may be an adaptation to the environments colonized by the Bacteroidales, including the mammalian gut.

## Introduction

The gut microbiota is an ecosystem rich in microbial diversity that has the potential to encode for a wide range of metabolic activities that may impact on host health and/or development^1–4^. Many activities associated with the gut microbiota are likely to be novel and may not be predicted by normal *in silico* DNA sequence analysis. Therefore, functional metagenomics can be used as a tool to identify novel activities in the microbiota. In this way several important and novel microbial activities that impact on host physiology have been identified^5,6^.

Cholesterol is an important structural component of mammalian cell membranes and it is either synthesised “de novo” in the body or it is acquired from dietary sources. Cholesterol metabolism is very important for eukaryotes as it is a precursor for vitamins, bile acids, and steroid hormones. However, high serum cholesterol is also an important risk factor for developing Coronary Heart Disease, a leading cause of death worldwide. Although the human gut microbiota can affect the physiology of the human host, the contribution of the gut microbiota to the metabolism of cholesterol is not well understood^7^. Germ-free C57BL/6 mice fed a high-fat diet had higher levels of cholesterol in their fecal pellets compared to conventional mice on the same diet suggesting that the presence of a microbiota can reduce the levels of cholesterol excreted in the feces^8^. However, a specific role, if any, for the microbiota in cholesterol metabolism is still not clear. Some members of the gut microbiota have been shown to have cholesterol reductase activity resulting in the reduction of cholesterol to coprostanol^9–12^. For example a strain of *Bacteroides* sp. was isolated from human feces and was shown to be able to reduce cholesterol to coprostanol, although the gene encoding this activity was not identified^13^.

In this study we have screened a functional metagenomic library constructed from the microbial DNA isolated from the feces of a healthy human male volunteer for activities that affect cholesterol solubilisation. In total 14 clones were identified and all of these clones mapped to the same 9-gene DNA fragment with strong homology to the genome of *Bacteroides vulgatus*, a member of the healthy human gut microbiota^14^. Further molecular analysis identified a single gene from this region that was responsible for the observed activity and this gene has been called *choA*. The *choA* gene has recently been shown to encode an amino acid *N*-acyltransferase that produces *N*-acyl-3-hydroxy-palmitoyl glycine or commendamide^15^. In this study we show that the production of commendamide is responsible for the observed cholesterol solubilizing activity and we report that commendamide also has strong hemolytic activity. We report the identification of amino acid residues that are important for ChoA activity and we present functional data to show that the phylogenetic distribution of *choA* is limited to the order Bacteroidales, a group of bacteria associated with the mammalian gut. This suggests that ChoA activity may be particularly relevant to life in this environmental niche.

## Results

### Isolation of fosmids with potential cholesterol-solubilizing activity

**Figure 1:**
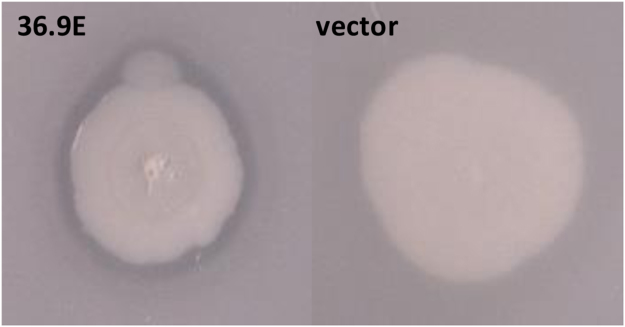
Halo production on LBC agar. EPI100 cells carrying a fosmid from a human gut metagenomic library were screened on LB agar containing 0.01% (w/v) cholesterol for the production of a halo, indicative of the presence of a gene(s) on the fosmid that encodes a factor that has activity against cholesterol. EPI100 carrying fosmid 36.9E is shown as an example of the type of halo observed.

The solubilisation of cholesterol is important for normal recycling and uptake with bile salts and phospholipids playing an important emulsifying role in mediating cholesterol solubility and reabsorption *in vivo* ^16^. To determine what role, if any, the gut microbiota might have in cholesterol metabolism we screened a metagenomic library for encoded activities that might contribute to the solubilisation of cholesterol. The metagenomic library was constructed from microbial DNA isolated from the fecal sample of a heathy male human volunteer and the library was maintained in the heterologous host, *E. coli* EPI300. LB agar becomes turbid after the addition of 0.01% (w/v) cholesterol (through the formation of cholesterol micelles or crystals)^17,18^. Therefore, we screened our metagenomic library for DNA that encoded proteins which affected the formation of these micelles. Individual *E. coli* clones containing fosmids were picked onto LB agar plates containing 0.01% (w/v) cholesterol (LBC agar) and, after incubation for 48 h at 37 °C, each colony was scored for the presence/absence of a zone of clearing around the bacterial colony. In total 41,472 clones (average insert size = 35kbp) were screened (1,451Mbp of microbiome DNA in total) and 21 clones, that produced a zone of clearing around the bacterial colony, were identified (see Fig. 1). The presence of a zone of clearing was considered indicative of the production of potential cholesterol-solubilizing activity by a gene(s) expressed from DNA present in the fosmid clone.

### Identification of *choA*

**Figure 2:**
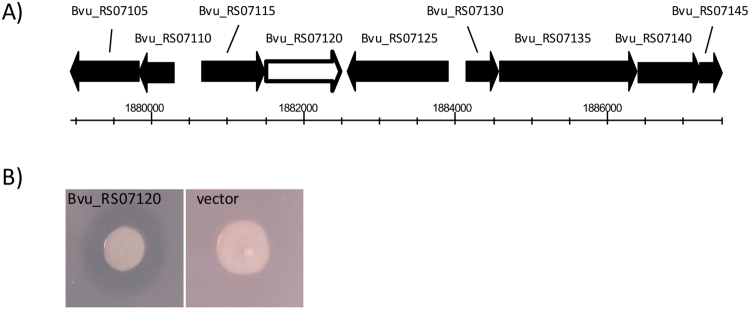
Identification of the gene required for the production of the halo on LBC agar. (**A**) Fosmid 36.9E was subjected to *in vitro* transposon mutagenesis and mutants that did not produce a halo on LBC agar were identified. Sequencing identified that the transposons in these mutants had inserted into a gene with homology to Bvu_RS07120 from *Bacteroides vulgatus*. (**B**) The Bvu_RS07120 gene was cloned into pTRC99a, transformed into EPI100 and tested for the production of a halo on LBC agar.

The DNA fragment present in 14 out of the 21 clones was identified by end-sequencing of the fosmid insert and BLAST analysis. All of the sequenced DNA fragments exhibited strong homology (>99% identity) to the same region of genomic DNA from *Bacteroides vulgatus* ATCC8482 predicted to contain the ORFs, Bvu_RS07105 to Bvu_RS07145 (see Fig. 2). Bvu_RS07105 and Bvu_RS07110 are predicted to encode proteins with homology to rRNA small subunit methyltransferase H and MraZ respectively. Bvu_RS07115 is predicted to encode a glycerol acyltransferase and Bvu_RS07220 is annotated as a hemolysin. Bvu_RS07125 and Bvu_RS07130 are predicted to encode proteins involved in nucleotide metabolism, dGTP triphosphohydrolase and dUTP nucleotidohydrolase, respectively. Bvu_RS07135 and Bvu_RS07140 are predicted to encode hypothetical proteins and Bvu_RS07145 is predicted to encode EnvC, a septal ring factor involved in the activation of murein amidases. In an unbiased approach to link the cholesterol-solubilizing activity with a specific gene in this region we selected a fosmid (labelled 36.9E) and undertook *in vitro* random transposon mutagenesis. The mutated fosmid pool was electroporated into EPI300 cells and 2 mutants (out of a total of 960 mutants screened) that were unable to produce the zone of clearing on LBC agar were identified. The site of transposon insertion in these mutants was identified by DNA sequencing and was shown to be within Bvu_RS07120 (see Fig. 2A). The Bvu_RS07120 gene was cloned into pTRC99a (under the control of the IPTG-inducible p*trc* promoter) and expression of Bvu_RS07120 resulted in a zone of clearance when EPI300 cells containing the plasmid were cultured on LBC agar plates (see Fig. 2B). Therefore, we have identified an ORF from *B. vulgatus*, Bvu_RS07120, that is linked with the solubilisation of cholesterol and we have renamed the gene *choA* (and the plasmid carrying *choA* was named pTRC-*choA*).

### ChoA produces a bioactive amine that solubilises cholesterol

**Figure 3:**
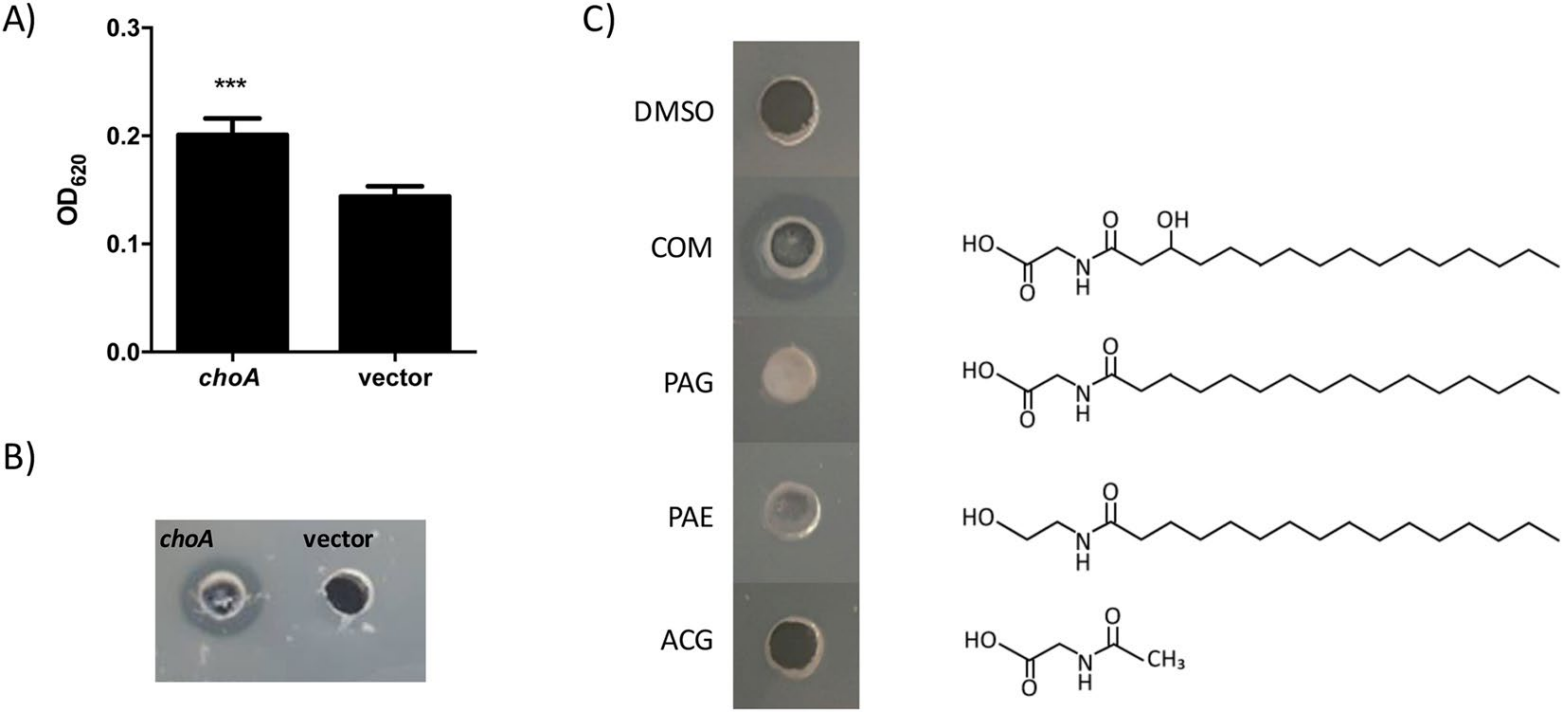
Commendamide activates NF-kB and produces a halo on LBC agar. (**A**) The cell-free supernatant (CFS) of *E. coli* EPI300 carrying pTRC-*choA* or the vector control were extracted with ethyl acetate and applied to monolayers of a HEK293 cell containing the SEAP reporter gene under the control of a NF-kB sensitive promoter. The level of SEAP expression is greater from cells treated with CFS from pTRC-*choA* expressing bacteria as compared to the pTRC99a vector. The experiment was carried out in triplicate and the error bars represent the standard deviation. (***P < 0.001). (**B**) A 50 μl aliquot of a crude ethyl acetate extract of the CFS from *E. coli* containing the pTRC99a-*choA* plasmid or the vector control was added to a well that had been made in a LBC agar plate. The plate was incubated at room temperature and the photograph was taken after 12 h (although the halo was seen to form within 1–2 h). (**C**) Synthetic commendamide produces a halo on LBC agar. Therefore 500 µg (50 µl of a 10 mg ml^−1^ solution) of synthetic commendamide and similar compounds were added to a well in a LBC agar plate and incubated at 37 °C for 48 h before the presence/absence of a halo was determined. DMSO: carrier control; COM: commendamide; PAG: N-palmitoylglycine; PAE: palmitoylethanolamide; ACG: N-acetylglycine. This experiment was repeated at least 3 times and a representative experiment is shown here.

To determine whether ChoA had an enzymatic activity that directly affected the solubility of cholesterol we purified the ChoA protein using a N-terminal 6XHis tag. The 6His-ChoA protein was overproduced and purified using Ni-affinity chromatography. The application of the purified protein directly on to LBC agar plates did not produce a zone of clearing suggesting that ChoA does not directly modify cholesterol (see Supplementary Figure S1). An alternative possibility is that ChoA produces a molecule that affects the solubility of cholesterol. Interestingly *choA* was recently described in an independent functional metagenomic screen looking for genes in the human gut microbiome that produced LPS-independent activators of NF-kB^15^. In this study the authors identified *choA* (called Cbeg12) as a gene encoding an amino acid acyltransferase that produces the bioactive amine, *N*-acyl-3-hydroxy-palmitoyl glycine or commendamide. Commendamide was shown to activate NF-kB through the G-protein coupled receptor, GPCR G2A/132^15^. To confirm that *choA* is producing commendamide in our system, cell-free supernatants (CFS) of cultures of *E. coli* carrying either the pTRC99a vector or the pTRC-*choA* plasmid were applied to a HEK293 cell line carrying a SEAP reporter gene under the control of a NF-kB sensitive promoter. As seen in Fig. 3A there is a significant increase in SEAP expression when the CFS from *E. coli* cultures expressing *choA* is added to the cell line compared to the CFS of *E. coli* cultures carrying the pTRC99a vector thus confirming the link between *choA* and NF-kB activation (see Fig. 3A). Moreover, the application of an aliquot of the ethyl acetate extract of CFS from cultures of *E. coli* carrying the pTRC99a-*choA* plasmid resulted in a zone of clearing on LBC agar plates (see Fig. 3B). Analysis of the ethyl acetate extract using high-resolution mass spectrometry also identified a peak, specific for the pTRC-*choA* extract, with a predicted mass that was similar to commendamide (see Supplementary Figure S2). We wanted to confirm that the production of commendamide by ChoA was responsible for the zone of clearing observed on LBC agar plates. To address this, we added 50 μl of a 10 mg ml^−1^ solution of synthetic commendamide (>95% purity as determined by NMR) to a well in a LBC agar plate. A zone of clearing, indicating cholesterol solubilisation, was observed around the well containing commendamide (see Fig. 3C). Importantly, the DMSO carrier and other acylated compounds tested (N-acetylglycine (ACG), N-palmitoylethanolamide (PAE) and N-palmitoylglycine (PAG)) did not produce zones of clearing when applied to LBC agar plates indicating that the activity of commendaminde on LBC agar is specific. Therefore, our data confirms that ChoA produces a molecule, *N*-acyl-3-hydroxy-palmitoyl glycine or commendamide, that has the ability to solubilize cholesterol.

### ChoA is distributed throughout the *Bacteroidales*

**Figure 4:**
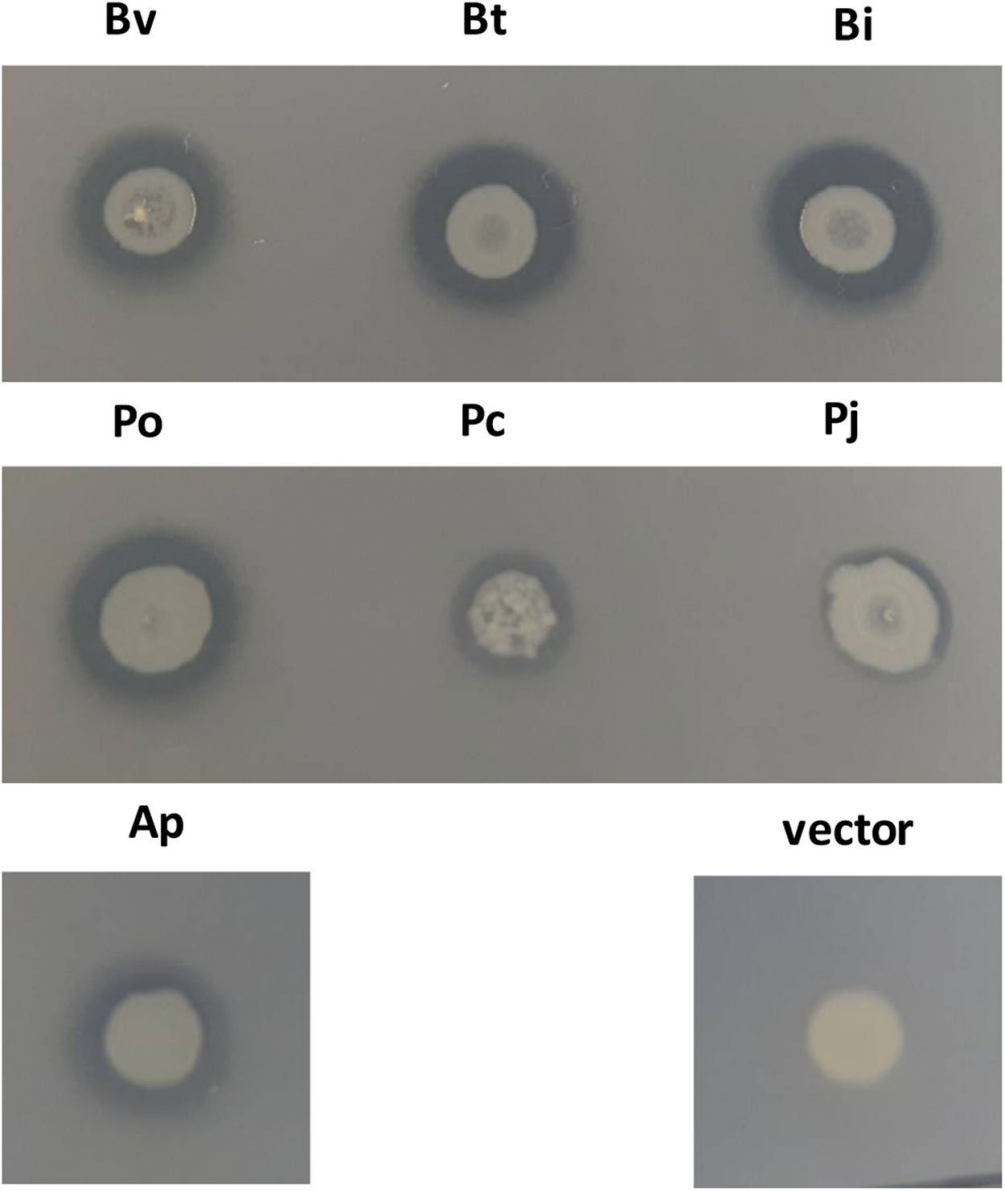
ChoA orthologues from across the Bacteriodales are associated with cholesterol solubilisation*. E. coli* EPI300 expressing different orthologues of *choA* were grown on LBC agar at 37 °C for 48 h. An orthologue was scored positive for commendamide production if there was a halo of any size observed around the colony. As a comparison there is no halo around the vector control colony. Bv: *Bacteroides vulgatu*s (YP_001298717.1); Bt: *Bacteroides thetaiotamicron* (NP_812371.1); Bi: *Bacteroides intestinali* (ZP_03013295.1)*s*; Po: *Prevotella oralis* (WP_004369353.1); Pc: *Porphyromonas catonaie* (WP_044167815.1); Pj: *Parabacteroides johnsoni* (WP_008156608.1); Ap: *Alistipes putredinis* (WP_004329950.1); vector: pTRC99a.

To determine the phylogenetic distribution of ChoA we undertook TBLASTN analysis of the bacterial genomes available at NCBI using the predicted amino acid sequence of ChoA from *B. vulgatus* as our query. Potential orthologues (with E vales < 10^−21^) were found in genomes from *Bacteroides* (taxaid: 816), *Prevotella* (taxaid: 838), *Porphyromonas* (taxaid: 836), *Parabacteriodes* (taxaid: 375288), *Alistipes* (taxaid: 239759) and *Flavobacterium* (taxaid:237). Orthologues were also identified distributed sporadically in the Proteobacteria e.g. *Vibrio harveyi*. Comparison of the predicted amino acid sequences confirmed the presence of ChoA orthologues in *Bacteroides thetaiotamicron* VPI5482 (BT_3459), *Bacteroides intestinalis* DSM17393 (BACINT_RS03885), *Parabacteroides johnsonii* DSM18315 (PARABACJOHN_RS00935), *Prevotella oralis* ATCC33269 (HMPREF0663_RS05760), *Porphyromonas catoniae* ATCC51270 (HMPREF0636_RS00930) and *Alistipes putredinis* DSM17216 (ALLIPUT_RS10580) (see Table 1). All of these bacteria are members of the Bacteroidales. The ChoA orthologues in *Flavobacterium* and *V. harveyi* were longer than the query sequence (587 and 604 amino acids in *V. harveyi* and *Flavobacterium*, respectively, compared to 323–332 amino acids in the Bacteroidales) and had only 25.33% and 25.17% amino acid identity with ChoA from *B. vulgatus*, respectively (see Table 1). Therefore, our data suggests that the *choA* gene is restricted to the Bacteroidales. Indeed, when expressed in *E. coli*, all of the *choA* orthologues cloned from the Bacteroidales produced a halo on LBC agar plates suggesting the production of commendamide, although the halos associated with the orthologues from *P. johnsonii* and *P. catoniae* are clearly smaller in size (see Fig. 4).

**Table 1:** Percent identity matrix for ChoA orthologues.

Strain*	Ap	Pc	Pj	Po	Bv	Bt	Bi	Fl	Vh
Ap	100	52.38	52.09	50.16	48.40	51.24	52.08	24.66	30.34
Pc	52.38	100	59.01	55.66	57.98	58.05	56.75	25.41	27.42
Pj	52.09	59.01	100	60.87	66.15	65.22	64.29	25.67	25.17
Po	50.16	55.66	60.87	100	74.54	70.43	74.70	25.91	26.51
Bv	48.40	57.98	66.15	74.54	100	77.61	81.60	25.33	25.17
Bt	51.24	58.05	65.22	70.43	77.61	100	82.32	26.67	27.52
Bi	52.08	56.75	64.29	74.70	81.60	82.32	100	26.33	25.84
Fl	27.00	25.41	25.67	25.91	25.33	26.67	26.33	100	34.08
Vh	30.34	27.42	25.17	26.51	25.17	27.52	25.84	34.08	100

*Ap: Alistipes putredinis; Pc: Porphyromonas catonaie; Pj: Parabacteroides johnsoni; Po: Prevotella oralis; Bv: Bacteroides vulgatus; Bt: Bacteroides thetaiotaomicron; Bi: Bacteroides intestinalis; Fl: Flavobacterium and Vh: Vibrio harveyi.

### Structure-function analysis of ChoA

**Figure 5:**
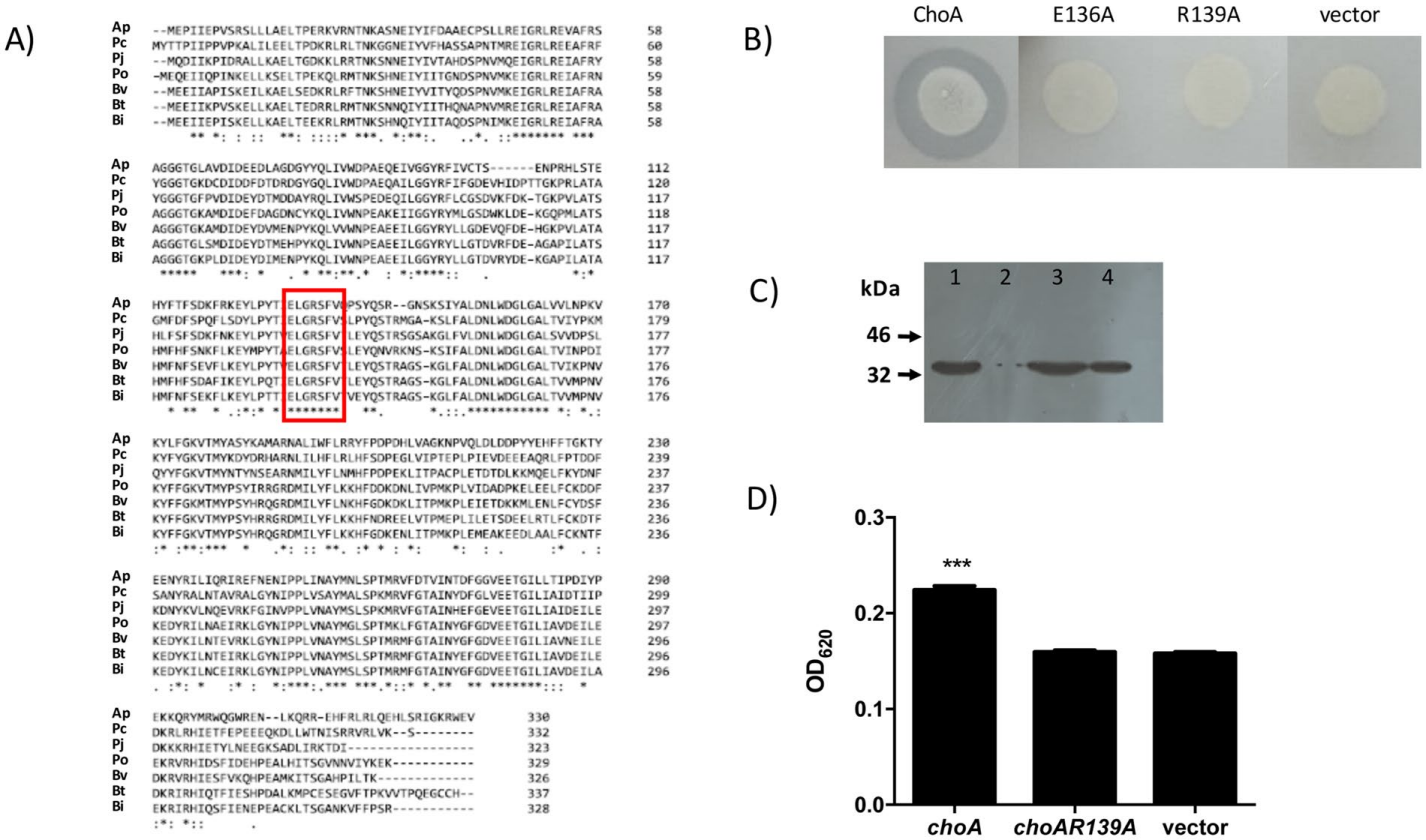
The identification of amino acids important for the activity of ChoA. (**A**) Clustal analysis of ChoA orthologues from the Bacteroidales. The conserved region discussed in the text is highlighted by the box and species annotations are as indicated in in Fig. 4. (**B**) Activity of EPI300 cells expressing *choA* and mutant derivatives on LBC agar plates. Plates were inoculated with the appropriate bacteria and incubated at 37 °C for 48 h. (**C**) Immunoblot analysis of total cell extracts from overnight cultures of *E. coli* EPI300 containing (1) pTRC99a-*choA*, (2) pTRC99a, (3) pTRC-*choAE136A* and (4) pTRC-*choAR139A*. (**D**) The cell-free supernatant (CFS) of *E. coli* EPI300 expressing *choA*, *choAR139A* or the vector control were extracted with ethyl acetate and applied to monolayers of a HEK293 cell containing the SEAP reporter gene under the control of a NF-kB sensitive promoter. The experiment was carried out in triplicate and the error bars represent the standard deviation. (***P < 0.001).

Phyre^2^ analysis produced a model for ChoA (confidence = 99.7%) based on the structure of LasI, an autoinducer synthetase from *Pseudomonas aeruginosa* (LasI; PDB entry 1ro5)^19^. LasI is an acyl-CoA *N*-acyltransferase that is required for the synthesis of the *N*-acyl homoserine lactone signaling molecule involved in quorum sensing^20^. The structural homology between LasI and ChoA extends from residues 29–184 of ChoA indicating that the active site required for acyltransferase activity is likely to be localized to this region. ClustalW analysis of the different ChoA homologues from the Bacteroidales reveals several blocks of conserved amino acids in this region including the 136-ELGRSFV-142 motif (see Fig. 5A). A similar motif has recently been shown to be required for the activity of the COG3176 family of *N*-acyltransferases^21^. To test the functionality of this motif in ChoA we undertook site-directed mutagenesis whereby Glu136 (E136) and Arg139 (R139) were changed to Ala. The mutated *choA* genes were cloned into pTRC99a and expressed in EPI300 cells and plated on LBC agar. As can be seen in Fig. 5B both the E136A mutation and the R139A mutation completely abolished the production of a zone of clearing on LBC agar confirming a role for these amino acids in the activity of ChoA. Importantly neither mutation had any effect on the stability of the ChoA protein as determined by immunoblotting (see Fig. 5C). We also tested the CFS from cells expressing the *choA R139A* mutant for the activation of NF-kB and confirmed that the level of commendamide produced is reduced to below the detection limit of this assay (see Fig. 5D). Therefore, we have identified 2 amino acids, conserved in all ChoA orthologues, that are required for the production of commendamide thus defining part of the active site of this protein.

### ChoA has hemolytic activity

**Figure 6:**
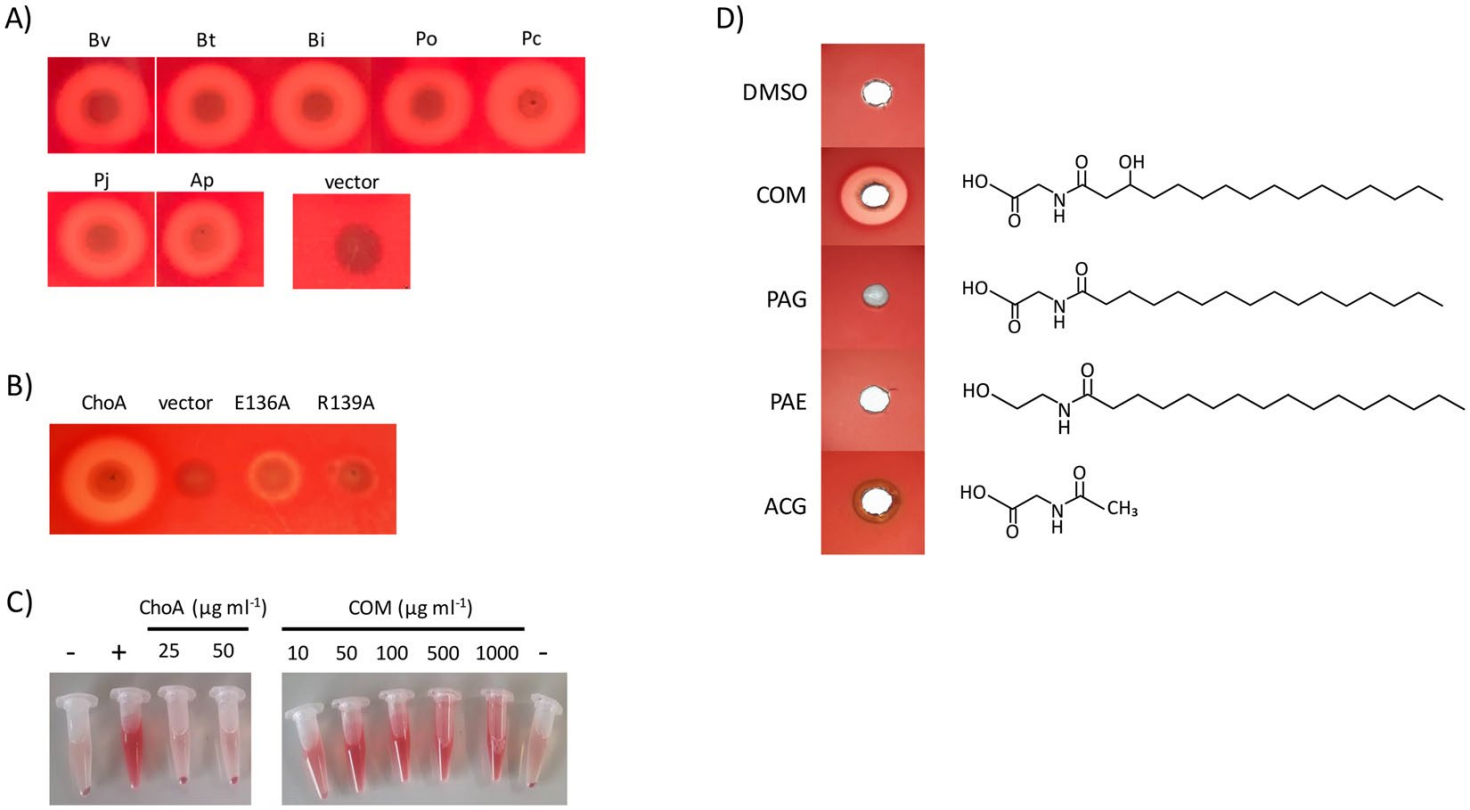
Commendamide has haemolytic activity. (**A**) EPI300 expressing different orthologues of *choA*, screened on horse blood agar. Bv: *Bacteroides vulgatu*s; Bt: *Bacteroides thetaiotamicron*; Bi: *Bacteroides intestinalis*; Po: *Prevotella oralis*; Pc: *Porphyromonas catonaie*; Pj: *Parabacteroides johnsoni*; Ap: *Alistipes putredinis*; vector: pTRC99a. (**B**) Hemolytic activity of EPI300 cells expressing *choA* and mutant derivatives as determined using horse blood agar. Agar plates were inoculated with the appropriate bacteria, cultured at 37 °C for 48 h before the presence/absence of a halo was scored. (**C**) Purified ChoA and commendamide (COM), at the indicated concentrations, was added to 1 ml of 2% (v/v) defibrinated horse blood in PBS and hemolysis was measured after 4 h incubation at 37 °C. Samples were compared to positive (blood cells lysed with water) and negative (no protein for ChoA and DMSO for COM) controls. (**D**) Commendamide and other acylated compounds were tested for hemolytic activity on horse blood agar. Therefore, 500 µg (50 µl of a 10 mg ml^−1^ solution) of each compound was added to a well in a horse blood agar plate and incubated at 37 °C for 48 h before the presence/absence of a halo was determined. DMSO: carrier control; COM: commendamide; PAG: N-palmitoylglycine; PAE: palmitoylethanolamide; ACG: N-acetylglycine. This experiment was repeated 3 times and a representative experiment is shown here.

In *B. vulgatus* the *choA* gene (Bvu_RS07120) is annotated as a putative hemolysin and the ChoA orthologue in *Bacteroides fragilis* has been reported to have hemolytic activity^22,23^. To confirm the hemolytic activity of ChoA, we plated *E. coli* EPI300 cells containing pTRC99a and the clones expressing the different *choA* orthologues on LB agar plates incorporating 5% (v/v) horse blood. After 48 h incubation at 37 °C a clear zone of hemolysis was observed around colonies expressing all of the *choA* orthologues except the vector control (see Fig. 6A). We also tested ChoA E136A and ChoA R139A for hemolytic activity and it is clear that hemolysin activity is greatly reduced/absent from these mutants confirming that these residues have an important role in the activity of ChoA (see Fig. 6B). Moreover, we could not detect any hemolytic activity when 50 μg ml^−1^ of the purified ChoA protein was incubated with horse blood in PBS (see Fig. 6C). Using the same assay, we could measure hemolysis (at a level of 49% total hemolysis) when commendamide was added at a concentration of 10 μg ml^−1^ and 100% hemolysis at all concentrations >10 μg ml^−1^ (see Fig. 6C). Finally, commendamide was tested using blood plates and a clear zone of hemolysis could be detected around the well (see Fig. 6D). In contrast similar acylated molecules did not produce a zone of clearing around the well suggesting that the potent hemolytic activity observed was specific to commendaminde (see Fig. 6D). Therefore, we propose that the hemolytic activity associated with ChoA is due to the production of commendamide.

## Discussion

Commendamide was previously identified as a molecule, produced by *Bacteroides* spp., that activates NF-kB through the GPCR protein G2A/132^15^. In this study we have confirmed these observations but we have also shown that commendamide production results in a halo on LB agar incorporating 0.01–0.02% (w/v) cholesterol (LBC agar plates) and hemolysis on blood agar plates. LBC agar plates have a turbid appearance and this turbidity is due to the formation of cholesterol micelles. Cholesterol has been shown to self-associate into micelles in aqueous solutions and the critical micellar concentration of cholesterol in aqueous solutions is 25–40 nM^18,24^. In this study we routinely use concentrations of cholesterol ranging between 0.01–0.02% (w/v) (equivalent to approximately 250–500 μM) in the LBC agar suggesting that cholesterol is forming micelles in our system. Therefore, we propose that commendamide production results in the solubilisation of these cholesterol micelles resulting in the observed halo. In addition, commendamide can also lyse red blood cells, an activity that we have shown is genetically linked to cholesterol solubilisation, and it is likely that hemolysis is a result of the solubilisation of the red blood cell membrane. However, it is not yet known whether the solubilisation activity of commendamide is specific for cholesterol and this is currently being investigated in the laboratory.

The Bacteroidales contains a number of bacterial species (e.g. *Bacteroides, Alistipes*) that represent a significant fraction of the human gut microbiota. Indeed, the *Bacteroides* genus has been identified as the most abundant genus in the healthy gut microbiota^14,25^. Therefore, as *choA* is found only in the Bacteroidales, ChoA activity may represent an important function in the human gut. ChoA is a member of the long-chain *N*-acyl amino acid synthase (NAS) group of enzymes^15,26^. Functional metagenomics screens of environmental DNA have identified a number of NAS proteins that have been shown to add long-chain acyl groups to different amino acids e.g. NasW acylates tryptophan, NasR acylates arginine, NasP acylates phenylalanine and NasY1 acylates tyrosine^26–28^. Interestingly, the occurrence of NAS activity is also thought to be a common phenomenon amongst environmental metagenomic libraries^28^. However, a Clustal Omega alignment of ChoA, NasY1, NasR, NasP and NasW revealed very little pairwise homology (<25% identity) suggesting that these enzymes do not share a common ancestor (see Supplementary Table S2). It has not yet been determined whether different acylated amino acids have the same activity as commendamide. However, our data does indicate that both cholesterol solubilisation and hemolysis requires the 3-hydroxy substitution present on the palmitoyl chain attached to the glycine as we have shown that N-palmitoylglycine is inactive (see Figs 3 and 6). Therefore, commendamide activity does not appear to be commonly associated with acylated amino acids.

It should be noted that all of the commendamide phenotypes reported here and elsewhere^15^ are associated with the heterologous overexpression of *choA* in *E. coli*. Therefore, the physiological role of *choA* in the Bacteroidales is still unknown. Commendamide has been detected in the supernatants of *Bacteroides* cultures after an extended stationary phase suggesting that this molecule may be secreted^15^. One possible function of commendamide may be to contribute to the solubilisation of cholesterol in the host in order to aid reabsorption in the gut^29^. Moreover, the timing of commendamide production suggests that this molecule may contribute to the adaptation of *Bacteroides* to starvation. Indeed, it was shown that a deletion mutant in *B. fragilis* that encompassed both the *hlyB* and *choA* homologues in this bacterium has a very strong growth phenotype^22^. Alternatively, we have noted that the structure of commendamide resembles that of the AHL molecules used during quorum sensing in many Gram negative bacteria. ChoA does have sequence, and predicted structural, homology with the enzymes known to produce the quorum-sensing AHLs (i.e AHL synthases) and it has been suggested that acylated amino acids may function as quorum sensing-like signals in some bacteria^26^. AHLs diffuse across the bacterial membranes in order to accumulate in the environment and it is possible that commendamide secretion may employ a similar mechanism. However, some acylated amino acids have been shown to integrate into the membrane bilayer in order to replace phospholipids during periods of phosphate limitation^30,31^. Therefore, it is also possible that the primary reservoir of commendamide molecule is the bacterial membrane and commendamide release may be a consequence of cell lysis.

Genome analysis indicates that *choA* is always found immediately downstream from another gene, predicted to encode a member of the lysophospholipid acyltransferase (LPLAT) superfamily (CDD:302626)^22^ (see Supplementary Figure S3). Moreover, the longer ChoA orthologues from *V. harveyi* and *Flavobacterium* have homology to LPLAT at the N-terminus with homology to ChoA at the C-terminus suggesting that these activities may be coupled (see Supplementary Figure S3). In *Sinorhizobium meliloti* the production of ornithine lipid (OL) has been shown to require two acyltrasferases, OlsA and OlsB. OlsB catalyses the first step in OL biosynthesis, the addition of a 3-hydroxy fatty acyl group to the α-amino group of ornithine resulting in the formation of lyso-OL^32^. OlsA has LPLAT activity that transfers a fatty acyl group to the 3-hydroxy group of the fatty acid in lyso-OL, thus forming the diacylated OL^33^. In contrast, *Serratia proteamaculans* uses a single bifunctional protein, with both LPLAT and acyltransferase activities, to produce OL^34^. Interestingly lyso-OL has been reported to have hemolytic activity (although we have not tested whether lyso-OL can solubilize cholesterol) whilst the diacylated OL is not hemolytic but does have haemagglutination activity^35^. Therefore, we propose that, in the Bacteroidales, ChoA is required for the production of commendamide (a lyso-glycine lipid) and the acyltransferase encoded by *hlyB* may conjugate a fatty acyl group to the hydroxyl-group on the palmitoyl moiety of commendamide to produce glycine lipid (GL). However, the production of GL remains to be confirmed experimentally and further work on the role of commendamide and/or GL in *Bacteroides* is currently under way in the laboratory.

## Methods

### Bacterial Strains and growth conditions

*Bacteroides vulgatus* ATCC 8482 and *Bacteroides thetaiotaomicron* VPI 5482 were routinely cultured in Brain Heart Infusion media (Sigma-Aldrich), supplemented with hemin (5 μg ml^−1^), cysteine (0.1% (w/v)), and sodium bicarbonate (0.2% (w/v)), and grown under anaerobic conditions at 37 °C. *E. coli* EPI300 was grown, aerobically at 37 °C, in Lysogeny broth (LB), supplemented, where appropriate, with ampicillin (100 μg ml^−1^) and chloramphenicol (12.5 μg ml^−1^).

### Metagenomic screen

A human gut metagenomic library, previously constructed from the feces of a healthy male^6^, expressed in *E. coli* EPI300 was screened for clones that could produce a halo around a colony on LBC agar (i.e. LB agar supplemented with 0.01% (w/v) cholesterol (Sigma-Aldrich)). LBC agar has a turbid appearance due to the formation of insoluble cholesterol micelles. Fosmid clones from the library were spotted onto Q-trays containing LBC agar, using a Genetix QPIX2 colony picking robot, and incubated at 37 °C for 48 h. After incubation, clones were visually examined for the presence of a clear halo around a colony. All of the potential positive clones were re-streaked from the original library onto LBC agar plates to conform halo production. The DNA fragment in each fosmid was identified by end-sequencing using the T7 and M13R primers (GATC Biotech).

### *In vitro* Transposon Mutagenesis

In order to identify the gene(s) responsible for the observed activity one fosmid, 36.9E, was subjected to *in vitro* transposon mutagenesis using the EZ-Tn5™ Tnp Transposome™ Kit (Epicentre), according to the manufacturer’s instructions. Individual mutants were screened for activity and, from 960 mutants screened, 2 were identified (7.3 G and 8.11 C) that could no longer produce a halo on LBC agar. The exact location of the transposon insertion in these mutants was identified by DNA sequencing using the KAN-2 FP-1 and KAN-2 RP-1 forward and reverse (respectively) transposon-specific primers (Epicentre).

### Cloning *choA* orthologues and mutant construction

The different *choA* orthologues identified in this study were amplified by PCR and cloned under the control of the IPTG-inducible P*trc* promoter present in pTRC99a^36^. Primers used for cloning were purchased from MWG-Biotech, and are listed in Supplementary Table S1. Genomic DNA was isolated from *Bacteroides vulgatus* and *Bacteroides thetaiotamicroan* using GenElute Bacterial Genomic DNA Kit (Sigma-Aldrich). In all other cases genomic DNA was purchased from DSMZ. Site directed mutagenesis was carried out on the cloned *choA* gene from *B. vulgatus* using QuikChange® Site-Directed Mutagenesis Kit (Agilent), according to manufacturer’s instructions and mutations were confirmed by sequencing (GATC Biotech).

### Blood Plate Assays

For agar-based hemolysin assays Tryptic Soy agar was supplemented with 5% (v/v) defibrinated horse blood (Cruinn) and 20 μM IPTG, to induce expression of *choA*. Overnight cultures, expressing the relevant *choA* clone, were pelleted by centrifugation (1000 g, 5 mins, at room temperature), resuspended in PBS and adjusted to an OD_6000_= 1. An aliquot of 5 μl of each culture was spotted onto the blood plate (and LBC agar plate where appropriate), in biological triplicate, along with a negative control. The plates were incubated at 37 °C for 48 h, and visually inspected for hemolysis or halos.

### Liquid Hemolysin Assay

The hemolytic activity of purified ChoA and commendamide was assessed using a liquid hemolysin assay. Reaction mixtures containing 2% (v/v) defibrinated horse blood in PBS were set up and purified ChoA or commendamide was added to the required concentration. For the positive control (i.e. 100% hemolysis) red blood cells were lysed in water and for the negative control, PBS (carrier for the purified ChoA) or DMSO (carrier for the commendamide) was added to the reaction mixture. All reaction mixtures were at a final volume of 1 ml. The reactions were incubated at 37 °C for 4 hours, before the samples were centrifuged for 3 min at 1000 g to gently spin out any intact red blood cells. The release of haemoglobin was determined by measuring the OD_545_ of the supernatant. The % haemolysis was calculated using the following formula: [(OD_545_ of sample − OD_545_ of negative control)/(OD_545_ of positive control − OD_545_ of negative control)] × 100.

### Purification of ChoA protein

The *choA* gene, from *B. vulgatus*, was cloned into pTRC99a-HIS, a pTRC99a-based vector that adds a tag encoding a N-terminal 6xHis tag to the cloned gene. An overnight culture of *E. coli* EPI300 containing pTRC99a-6His-*choA* was diluted 1/100 into 50 ml LB broth, and grown to an OD_600_ of 0.5. At this point 100 μM IPTG was added to induce *choA* expression, and the culture was incubated at 37 °C for a further 3 h. The cells were harvested by centrifugation for 10 mins, 1500 g, at 4 °C and the cell pellets were re-suspended in 2.5 ml ice cold 100 mM Tris–HCl, 1 mM EDTA, pH8 with lysozyme (100 μg ml^−1^) and incubated on ice for 30 min. At this point 10 mM MgCl_2_ and 50 μg ml^−1^ DNase I were added and the suspension was left for a further 15 min on ice. The cells were then lysed by 3 x freeze thaw cycles (−80 °C to 37 °C) and the lysate was centrifuged at 15000 g for 15 min at 4 °C. The pellet was kept as the insoluble fraction and the supernatant (soluble fraction) was filtered through a 0.45 μM filter (Sarstedt). The HisTrap affinity column (GE Healthcare) was prepared by washing with 5 ml dH_2_O, and then 5 ml 1x buffer A (4 mM Tris-HCl, 100 mM NaCl, pH 7.9). The bacterial soluble fraction was passed through the column, and the flow through was collected. The column was washed with 8–10 ml wash buffer (buffer A plus 10 mM imidazole) and the wash flow through was collected. The 6HIS-ChoA protein was eluted in buffer A containing 50 mM Imidazole and collected in 5 × 1 ml fractions. Each fraction, along with insoluble, soluble, and flow through fractions, was loaded onto a 12.5% SDS-PAGE gel to visualise protein bands and protein concentration was determined by the Bradford assay (Sigma-Aldrich).

### Immunoblot Analysis

*E. coli* EPI300 cells expressing the different *choA* orthologues were grown overnight in LB broth with 20 µM IPTG. Cells were centrifuged and resuspended to an OD_600_ = 1 in PBS, as previously described. Cells were washed 2X in PBS, re-suspended in 100 μl of 1X SDS sample buffer before 10 μl of each sample was heated to 95 °C for 5 min and loaded onto a 12.5% SDS-PAGE gel. After separation, proteins were transferred to a nitrocellulose membrane, and the membranes were blocked in 1X TBS with 5% skim milk, for 1 h at room temperature, with gentle agitation. The membrane was washed 3 times in 1X TBS plus 0.05% Tween-20 (TBST) before primary rabbit anti-ChoA antibody (Biogenes) was added to the membrane (dilution 1:1000) and incubated overnight at 4 °C, with gentle agitation. The membrane was washed 3 times in 1X TBST and anti-rabbit secondary antibody conjugated with horse-radish peroxidase (HRP) (Sigma-Aldrich) was added (dilution 1:80,000) and incubated with the membrane for 1 h at room temperature. The membrane was washed in 1X TBST and the protein bands visualised using SuperSignal West Pico Chemiluminescent Substrate (Thermo Scientific), according to manufacturer’s instructions.

### Assay for NF-kB activity

*E. coli* cells, carrying the appropriate plasmid, were cultured for 24 h at 37 °C, with agitation, in the presence of 20 μM IPTG. The cells were removed via centrifugation, and the cell-free supernatant was filtered through 0.2μm filter (Sarstedt). HEK-Blue™ hMD2-CD14 cells (containing a copy of the secreted alkaline phosphatase (SEAP) gene under the control of an NF-kB regulated promoter) were maintained in cell culture medium consisting of Dulbecco’s Modified Eagle’s Medium (DMEM) containing 4.5 g l^−1^ glucose and 2 mM L-glutamine (Gibco). The media was also supplemented with 10% (v/v) fetal bovine serum (Gibco), 50 U ml^−1^ penicillin, 50 μg ml^−1^ streptomycin, 100 μg ml^−1^ Normocin (Invivogen), 200 µg ml^−1^ Hygromycin B Gold (Invivogen), and 100 µg ml^−1^ Zeocin (Invivogen). To start the assay 20 μl of the bacterial cell-free supernatant was added to a flat bottomed 96-well plate and 180 μl of a 2.8 × 10^5^ cells ml^−1^ suspension of HEK-Blue™ hMD2-CD14 cells, washed in PBS and resuspended in HEK-Blue™ Detection medium, was added to each well. The HEK-Blue™ Detection medium contains a chromogenic substrate for SEAP and NF-kB activity was determined by simply measuring the OD_620_ after incubating the cells at 37 °C for 12 h. Cell-free supernatants prepared from bacteria carrying the vector control were used as negative control.

### Mass spectrometry

The presence of commendamide in culture supernatants was confirmed using mass spectrometry. Bacterial cultures were grown, and induced with 20 μM IPTG, for 24 h at 37 °C and the cells were removed by centrifugation at 1500 g for 15 min at room temperature. An equal volume of ethyl acetate (Sigma-Aldrich) was added to the cell-free culture supernatant and the solution was mixed thoroughly before the organic fraction was collected, and dried under vacuum. The samples were resuspended in acetonitrile and low-resolution mass spectra were recorded on a Waters Quattro Micro triple quadropole instrument in electrospray ionisation (ESI) mode using 50% acetonitrile-water containing 0.1% (v/v) formic acid as eluent. High resolution precise mass spectra (HRMS) were recorded on a Waters LCT Premier Tof LC-MS instrument in electrospray ionisation (ESI) mode using 50% acetonitrile-water containing 0.1% (v/v) formic acid as eluent.

## Electronic supplementary material

Supplementary Information
