# Supplementary material for: The Bacteroidales produce an N-acylated derivative of glycine with both cholesterol-solubilising and hemolytic activity
Source: Sci Rep. 2017 Oct 16;7:13270. doi: 10.1038/s41598-017-13774-6 (PMC5643525; doi:10.1038/s41598-017-13774-6)
Supplement: Supplementary file 1 — Supplementary Information [file 41598_2017_13774_MOESM1_ESM.pdf]

Supplementary information for:

The Bacteroidales produce an *N*-acylated derivative of glycine with both cholesterol-solubilising and hemolytic activity.

Alli Lynch[1,2], Elaine Crowley[2], Eoghan Casey[2], Rafael Cano[3], Rachel Shanahan[3], Ger McGlacken[3], Julian R. Marchesi[4], David J. Clarke[1,2*]

[1]School of Microbiology, [2]APC Microbiome Institute, [3]Department of Chemistry, University College Cork, Ireland; [4]School of Biosciences, Cardiff University, Museum Avenue, Cardiff CF10 3AX, UK and Centre for Digestive and Gut Health, Imperial College London, London W2 1NY, UK

*Corresponding author

Email: david.clarke@ucc.ie

Phone: +353 (0)21 4903624

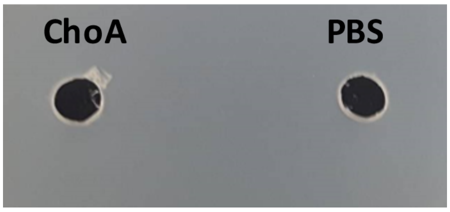

Supplementary Figure S1. Purified ChoA protein does not produce a halo on a LBC agar plate. An aliquot of 50µl of 0.5mg ml^{-1} purified N-terminal His-tagged ChoA protein (25ug protein in total) and 50µl of a PBS control, was added to a well in a LBC agar plate. The agar plate was sealed with parafilm and incubated for 24h at 37°C.

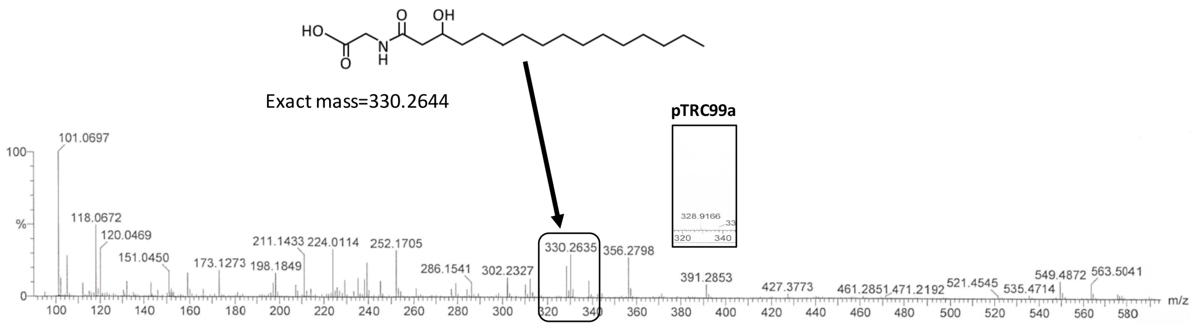

Supplementary Figure S2. Mass spectroscopy of cell-free culture supernatants from *E. coli* EPI300 with pTRC-*choA*. Bacterial cultures were grown, and induced with 20µM IPTG, for 24 h at 37°C and the cells were removed by centrifugation at 1500g, 15 min at room temperature. An equal volume of ethyl acetate (Sigma-Aldrich) was added to the cell-free culture supernatant and the solution was mixed thoroughly before the organic fraction was collected, and dried under vacuum. The samples were resuspended in acetonitrile and high resolution precise mass spectra (HRMS) were recorded on a Waters LCT Premier Tof LC-MS instrument in electrospray ionisation (ESI) mode using 50% acetonitrile-water containing 0.1% (v/v) formic acid as eluent. Compounds with a molecular mass very similar to the predicted mass of commendamide were detected in the supernatants from *E. coli* cells carrying pTRC-*choA* (as indicated) and were not observed in supernatants from *E. coli* cells carrying the pTRC99a vector (inset). The analysis was repeated with biological triplicate samples with identical results.

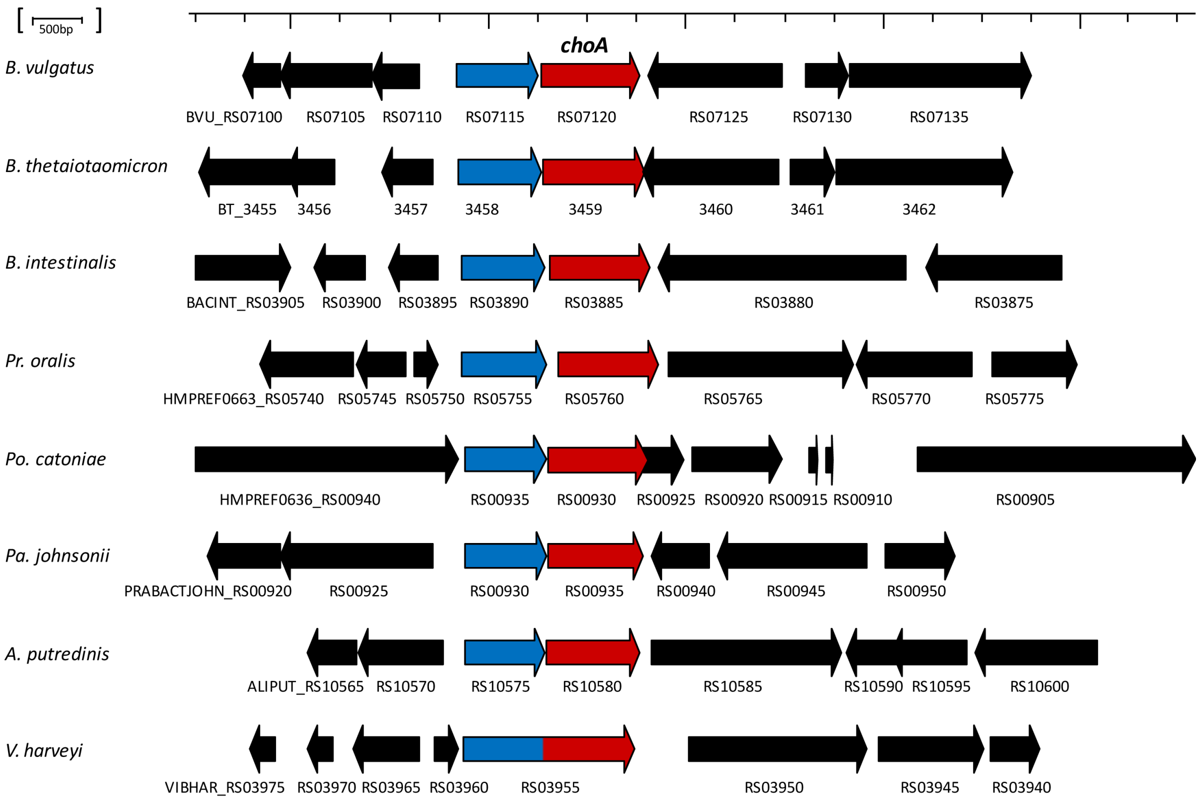

Supplementary Figure S3. Genomic context of *choA* in the different bacteria used in this study. Orthologues of *choA* (red) were always located immediately downstream from a gene encoding a protein with predicted LPLAT activity (blue). The ChoA orthologue in *V. harveyi* (VIBHAR_RS03955) appears to be a fusion of these proteins with predicted homology as indicated.

Supplementary Table S1. Primers used in this study.

Primer	Sequence 5'-3'	Use in study
FwchoA.Nco1	ctgctgCCATGGAAGAAATTATTGCACCGATTAGC	Cloning *choA* from *B. vulgatus* ATCC 8482
RvchoA.Xba1	ctgctgTCTAGATTATTTGGTCAGTATGGGATGA	Cloning *choA* from *B. vulgatus* ATCC 8482
FwThet.Nco1	ctgctgCCATGGAAGAGATTATTAAACCGGTGAG	Cloning *choA* from *B. thetaiotaomicron* VPI-5482
RvThet.Xba1	ctgctgTCTAGATTAATGGCAACAGCCTTCCTG	Cloning *choA* from *B. thetaiotaomicron* VPI-5482
FwPrev.SacI	ctgctgGAGCTCATGGAACAAGAGATTATTCAACC	Cloning *choA* from *Prevotella oralis* ATCC 33269
RvPrev.XbaI	ctgctgTCTAGATTACTTTTCCTTGTAGATTACATTA	Cloning *choA* from *Prevotella oralis* ATCC 33269
FwB.intest.NcoI	ctgctgCCATGGAAGAGATTATTGAACCTATAAGTA	Cloning *choA* from *B. intestinalis* DSM 17393
RvB.intest.XbaI	ctgctgTCTAGATCATCTACTTGGAAAGAAAACTT	Cloning *choA* from *B. intestinalis* DSM 17393
FwPara.SacI	ctgctgGAGCTCATGCAAGACATTATCAAACCGATT	Cloning *choA* from *Parabacteroides johnsonii* DSM 18315
RvPara.XbaI	ctgctgTCTAGATTAGATGTCGGTCTTGCGTATC	Cloning *choA* from *Parabacteroides johnsonii* DSM 18315
FwPor.EcoRI	ctgctgGAATTCATGTACACCACACCCATCATC	Cloning *choA* from *Porphyromonas catoniae* ATCC 51270
RvPor.XbaI	ctgctgTCTAGACTAGCTCTTCACCAATCGTAC	Cloning *choA* from *Porphyromonas catoniae* ATCC 51270
FwAlis.NcoI	ctgctgCCATGGAACCCATCATAGAGCCTGTAA	Cloning *choA* from *Alistipes putredinis* DSM 17216
RvAlis.XbaI	ctgctgTCTAGACTACACTTCCCATCTTTTCCC	Cloning *choA* from *Alistipes putredinis* DSM 17216
FwVIBhemo	ctgctgccATGGATAGTTCGACCCCTTTTCG	Cloning from *Vibrio harveyi* BB120
RvVIBhemo	ctgctggtcgacTTACTTATGGTTGCTATGTGC	Cloning from *Vibrio harveyi* BB120
FMutChoA(R-A)	CGGTGGAGTTGGGAGCCTCATTTGTTACATTG	Site Directed Mutagenesis (R139A)
RMutChoA(R-A)	CAATGTAACAAATGAGGCTCCCAACTCCACCG	Site Directed Mutagenesis (R139A)
FMutChoA(E-A)	CCTTACACGGTGGCGTTGGGACGCTC	Site Directed Mutagenesis (E136A)
RMutChoA(E-A)	GAGCGTCCCAACGCCACCGTGTAAGG	Site Directed Mutagenesis (E136A)

Supplementary Table S2. Percent identity matrix of ChoA, NasY1, NasW, NasP and NasR

	ChoA	NasY1	NasW	NasP	NasR
ChoA	100.00	19.91	16.76	19.73	17.13
NasY1	19.91	100.00	11.76	9.95	17.91
NasW	16.76	11.76	100.00	23.27	22.50
NasP	19.73	9.95	23.27	100.00	23.12
NasR	17.13	17.91	22.50	23.12	100.00
